# Experimental Assessment of the Effects of Temperature and Food Availability on Particle Mixing by the Bivalve *Abra alba* Using New Image Analysis Techniques

**DOI:** 10.1371/journal.pone.0154270

**Published:** 2016-04-26

**Authors:** Guillaume Bernard, Jean-Claude Duchêne, Alicia Romero-Ramirez, Pascal Lecroart, Olivier Maire, Aurélie Ciutat, Bruno Deflandre, Antoine Grémare

**Affiliations:** 1 Univ. Bordeaux, EPOC, UMR 5805, F33400, Talence, France; 2 Tvärminne zoological station, University of Helsinki, Hanko, Finland; 3 CNRS, EPOC, UMR 5805, F33400, Talence, France; Bangor University, UNITED KINGDOM

## Abstract

The effects of temperature and food addition on particle mixing in the deposit-feeding bivalve *Abra alba* were assessed using an experimental approach allowing for the tracking of individual fluorescent particle (luminophore) displacements. This allowed for the computations of vertical profiles of a set of parameters describing particle mixing. The frequency of luminophore displacements (jumps) was assessed through the measurement of both waiting times (i.e., the time lapses between two consecutive jumps of the same luminophore) and normalized numbers of jumps (i.e., the numbers of jumps detected in a given area divided by the number of luminophores in this area). Jump characteristics included the direction, duration and length of each jump. Particle tracking biodiffusion coefficients (D_b_) were also computed. Data originated from 32 experiments carried out under 4 combinations of 2 temperature (*Te*) and 2 food addition (*Fo*) levels. For each of these treatments, parameters were computed for 5 experimental durations (*Ed*). The effects of *Se*, *Fo* and *Ed* were assessed using PERmutational Multivariate ANalyses Of VAriance (PERMANOVAs) carried out on vertical depth profiles of each particle mixing parameter. Inversed waiting times significantly decreased with *Ed* whereas the normalized number of jumps did not, thereby suggesting that it constitutes a better proxy of jump frequency when assessing particle mixing based on the measure of individual particle displacements. Particle mixing was low during autumn temperature experiments and not affected by *Fo*, which was attributed to the dominant effect of low temperature. Conversely, particle mixing was high during summer temperature experiments and transitory inhibited by food addition. This last result is coherent with the functional responses (both in terms of activity and particle mixing) already measured for individual of the closely related clam *A*. *ovata* originating from temperate populations. It also partly resulted from a transitory switch between deposit- and suspension-feeding caused by the high concentration of suspended particulate organic matter immediately following food addition.

## Introduction

In aquatic environment, bioturbation may be defined as “all transport processes carried out by animals that directly or indirectly affect the sediment matrices” [1]. Such processes include both particle mixing and bioirrigation. Through bioturbation, benthic fauna strongly affects the chemical, physical and geotechnical properties of marine sediments [1, 2, 3, 4, 5, 6]. Particle mixing controls the transfer of recently settled particles to deeper sediment layers and thereby affects the remineralisation of particulate organic matter [7, 8, 9].

Particle mixing mainly results from locomotion, burrowing, defecation and feeding activities of the benthic macrofauna [10]. The effect of disturbance (and especially organic matter enrichment) on benthic community structure and processes (including bioturbation) is well documented [11, 12]. At the organism’s level, key environmental factors such as organic matter availability and water temperature are well known to tightly control the overall behaviour of benthic fauna; including burrowing and/or feeding activities [13, 14, 15] thereby altering particle mixing modes and rates [16, 17, 18, 19].

Particle mixing is classically quantified using particle tracers [20]. As opposed to natural ones (e.g. ^7^Be ^210^Pb, ^234^Th), which are naturally present in the sediment column, artificial tracers, such as luminophores (i.e. sediment particles with a fluorescent coating), are introduced at the sediment-water interface at the beginning of an experiment, and their vertical distribution within the sediment column is then measured after an incubation period of known duration. The observed vertical tracer profile is then described by fitting of a mathematical model. Several particle-mixing models are available. Due to its simplicity, the biodiffusive model [21, 22, 4, 23, 24, 25, 26, 27] has long been preferentially used despite the fact that its underlying hypotheses (i.e., highly frequent and very short isotropic jumps) are often not fulfilled [28]. In this model, particle mixing by benthic fauna is described by a single parameter: the biodiffusion coefficient. Recent years have seen the emergence of the continuous time random walk (CTRW) model [28, 29, 30, 31]. The CTRW model implements a stochastic description of particle mixing events. Particle displacement is then described as a random process, and each individual particle displacement is governed by three stochastic variables: (1) the jump direction, (2) the jump length, and (3) the waiting time between two consecutive jumps of the same individual particle [32]. Overall, the joined frequency distributions of these random variables form the “mixing fingerprint” of a benthic community or of a benthic organism [29]. It is also possible to compute a particle-tracking biodiffusion coefficient (D_b_) from those fingerprints [29, 32].

The CTRW model has already been successfully used with the bivalves *Abra ovata* and *A*. *nitida* [17], the polychaete *Nephtys* sp. [33], the amphipod *Corophium volutator* [34], or with natural communities [35]. In all these studies, mixing fingerprints were assessed: (1) assuming a perfect spatial homogeneity of particle mixing, (2), based on *a priori* assumed frequency distributions of waiting times and jump lengths and (3) through the fitting of a CTRW model to vertical luminophore profiles after a known period of incubation.

These points are questionable (see for example [29] for a discussion on the importance of the selection of *a priori* selected frequency distributions), explaining why Bernard *et al*. [36] have recently developed an experimental approach allowing for the direct and explicitly 2-D assessment of particle mixing fingerprints in the deposit feeding bivalve *Abra alba*. These authors adapted existing high frequency images acquisition and analysis techniques [16, 17, 18, 37, 38, 39] to track the motions of isolated luminophore along the wall of thin aquaria and to directly derive the frequency distributions of waiting times, jump lengths and directions. This allowed for the 2-D assessment of changes in particle mixing at a sub-millimetre resolution, which revealed the highly spatially heterogeneous behaviour of the particle mixing induced by *A*. *alba* under field-like conditions.

Particle mixing intensity in *A*. *alba* has previously been assessed trough the fit of the CTRW model to experimentally derived luminophore profiles [33] These authors reported a significant effect of water temperature but no significant effect of clam density on particle mixing. The effects of temperature [16, 17] and food availability [13, 16] on feeding activity and particle mixing have also been assessed in two closely related species: *A*. *ovata* and *A*. *nitida*. Corresponding results have shown: (1) differences in the functional responses (both in terms of feeding activity and particle mixing) of the two species to food addition, and (2) a significant effect of temperature on particle mixing in both species. In spite of a strict similarity in experimental procedures, there were important discrepancies between the studies carried out by Maire *et al*. [16, 17]. During the first study, these authors indeed studied the two *Abra* species and fitted a biodiffusive model to vertical luminophore profiles, whereas during the second one they worked with only *A*. *ovata* and fitted a CTRW model to vertical luminophore profiles. As underlined above, both approaches are no longer considered optimal in describing particle mixing. The aim of the present study was therefore to use the new image analysis techniques developed by Bernard *et al*. [36] to assess the effect of temperature and food availability on particle mixing in *A*. *alba*.

## Materials and Methods

### 2.1. Bivalve collection and maintenance

The deposit-feeding bivalve *Abra alba* (*Tellinacea*) is a dominant macrobenthic species in shallow subtidal areas along the European Atlantic coast [40, 41]. The body of this clam is usually buried a few centimetres deep in the sediment surface, while its siphons connect to the sediment-water interface. It reworks the upper sediment layer by protruding its inhalant siphon and aspiring recently deposited organic matter. Foraging movements consist of circular motions of the tip of the inhalant siphon at the sediment-water interface [42]. During the present study, sediment samples were collected in June 2010 and May 2011 in the Arcachon Bay (45°43’476 N, 1°37’758 W, 3–5 m depth) using a Van-Veen grab. No specific permissions were required and no endangered or protected species were affected by the sampling. Sediment samples were sieved through a 1 mm square mesh, yielding ~500 clams (9–12 mm in total shell length). Sediment grain-size was determined using a laser microgranulometer (MALVERN® Master Sizer S), whereas sediment Particulate Organic Carbon (POC) and Nitrogen (PON) contents were assessed using a CN auto-analyzer (Thermo Flash® EA112). Samples were freeze-dried prior to analyses and carbon analyses performed after decarbonatation (HCl 0.3 N). The sediment (47.7 ± 1.32% sand >63 μm and 52.3 ± 1.32% mud < 63 μm; 1.40 ± 0.07%DW (Dry Weight) POC and 0.16 ± 0.01%DW PON) was used both for maintenance of organisms and experiments. Clams were kept in tanks (60x40x30 cm) filled with field sieved (1 mm mesh) sediment and supplied with running ambient seawater (salinity: 32.5–34.8; temperature: 8.1–24.2°C) prior experimentation. During that period of time, they were fed once a week with crushed Tetramin® fish food.

### 2.2. Experimental set-up

**Fig 1:**
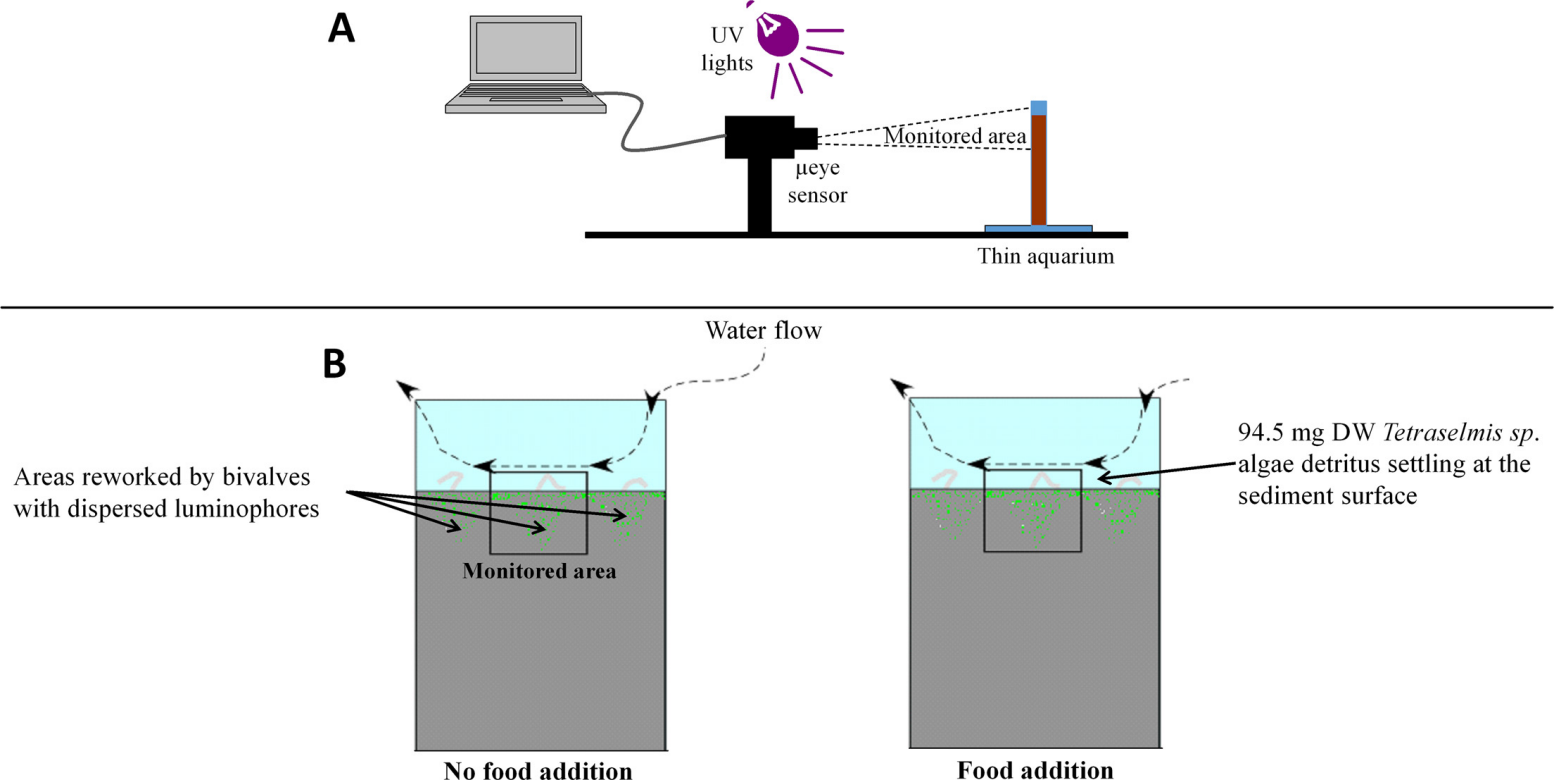
Experimental setup. Lateral view (A), Frontal views under the two conditions of food addition (B). Luminophores are in green.

The experimental set-up involved the use of thin aquaria, luminophores, UV lights, and high frequency image acquisition [16, 17, 36] (Fig 1). Thin aquaria (L = 17 cm, W = 0.9 cm, H = 33 cm) were filled with 15 cm of field sieved sediment. They were kept in a temperature-controlled climate room at ambient seawater temperature for 3 days before each experiment. Three bivalves of known size were then gently placed at the sediment surface, after which they typically buried within 30 seconds. If a clam did not do so within a minute, it was replaced. After 24 hours, 1.5 gDW of yellow luminophores (Geotrace Environmental Tracing®, median diameter = 35 μm) were spread at the sediment surface using a Pasteur pipette. Thin aquaria were placed 30 cm in front of two UV lights (which allowed for the distinction between fluorescent luminophores and the surrounding sediment particles) and of a μeye video captor (IDS®), which was positioned to monitor luminophore movements. The μeye captor had a definition of 2560x1920 pixels, while the monitored sediment area was 4.2 cm x 3.1 cm, which resulted in a resolution of 16.5 μm per pixel. Experiment began 24 hours after luminophore introduction. This allowed for: (1) the monitored field to be centred on an area effectively reworked by a single clam, and (2) the preliminary dispersion of luminophores. Each experiment lasted 48 hours and image frequency acquisition was 0.1 Hz. The series of images collected during each experiment were assembled in an AVI video format for further image analysis.

Here, we report on the results of 32 experiments during which both seawater temperature (two *Te* conditions) and Food addition (two *Fo* conditions) were manipulated in a balanced experimental design leading to 8 replicated experiments per combination of *Te* and *Fo* conditions. All experiments were carried out at ambient temperature. The “Summer” *Te* condition corresponded to experiments carried out between 17.6 and 22.3°C and the “Autumn” *Te* condition between 13.7 and 16.6°C. The “Without” *Fo* condition corresponded to experiments carried out in the absence of any organic input other that those contained in the running sea water used to supply the aquaria. Conversely, the “With” *Fo* condition corresponded to the introduction of 94.5 mgDW of fresh *Tetraselmis sp*. detritus (Marine-Life®, 6.03 ± 0.04% DW PON, 45.40 ± 0.36% DW POC) one hour prior the beginning of each experiment, leading to an enrichment of the sediment top 2 millimetres of ca. 43.9 and 37.8% for PON and POC, respectively. The amount of detritus added was set to correspond to a nitrogen daily food ration to standing biomass ratio of 0.5, which has been shown sufficient to support maximal daily weight specific growth rate in the opportunistic polychaete *Capitella capitata* sp.1 [43].

### 2.3. Image processing

**Fig 2:**
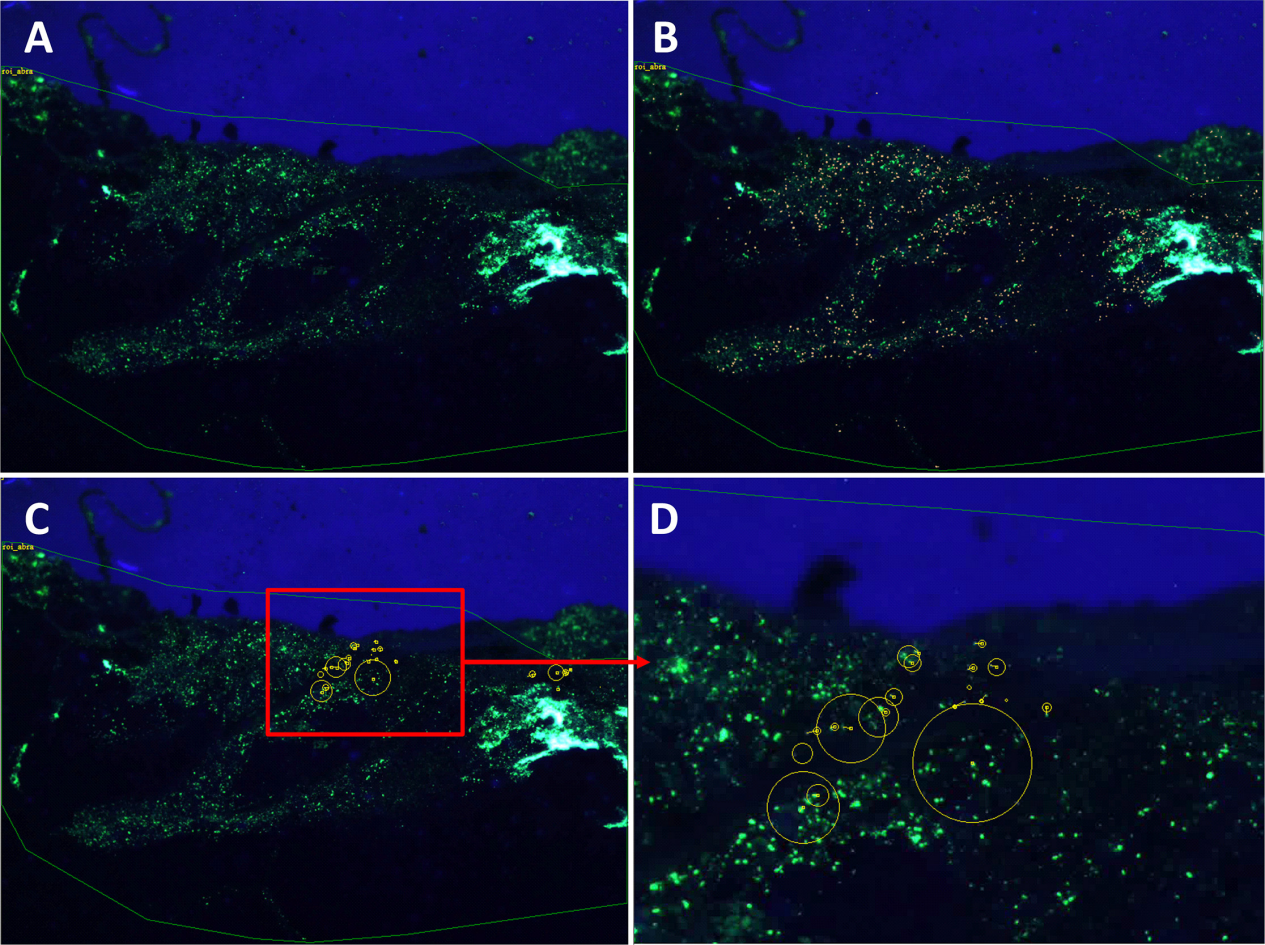
Example of visual outputs at the different steps of the image analysis process. Original image (A), localization of isolated luminophores (B), visualization (direction and length) of the jump vectors and of waiting times within the whole monitored plan (C), and zoomed for a specific area (D). Luminophores are in green (A, C, D). Isolated luminophores are in orange (B).Vector lengths in (C) and (D) are proportional to jump lengths. Circle diameters in (C) and (D) are proportional to waiting times.

For each experiment, AVI films were processed using two specific algorithms based on the analysis of the relative positions of isolated luminophore barycentres within consecutive images (S1 Software). These two algorithms are detailed in Bernard *et al*. [36]. Briefly, all isolated luminophores were first binarised for all images based on their green levels (threshold of 80 on a 0–255 scale) and apparent size (10–70 pixels). The (XY) coordinates of their barycentre in the pixel grid were then assessed for each individual image. The two algorithms respectively allow for the measurements of: (1) luminophore waiting time (i.e., the time lapses between two consecutive jumps of the same luminophore) and (2) luminophore jump characteristics (i.e., direction, duration and length). The first algorithm uses a single sensitivity circle centred on the luminophore barycentre, which accounts for both changes in the apparent size of luminophores due to fluctuations in UV light intensity, and to small movements of the sensor and/or the aquarium induced by vibrations. The second algorithm also uses a search circle that defines the maximum distance over which individual luminophores can be tracked between two consecutive images. Based on preliminary trials [36], the radius of the sensitivity and the search circles were set to 66 and 660 μm, respectively. When a jump event ended, the following parameters were recorded in the jump results file: the index number of the starting image (corresponding to the start of the jump), the index number of the final image (corresponding to the end of the trajectory analysis), the duration of the jump, the (XY) coordinates of the particle at the start of the jump, the (XY) coordinates at the end of the jump, the length and the direction of the jump vector. An example of visual outputs at these different steps is given in Fig 2.

### 2.4. Data processing

During each experiment, the location of the water–sediment interface was assessed based on its location within each 660 μm wide cell column defined (i.e., maximum Y coordinates within each cell column where waiting times were recorded). It was then translated to the first row of each cell column. After this operation, the cell Y-position in the picture thus corresponded to its depth within the sediment column [16]. We then computed 1D vertical 1320 μm resolution profiles of: (1) normalized numbers of jumps (the number of jumps detected in a given area during a given period of time divided by the number of luminophores in this area during this period of time), (2) mean inversed waiting time (1/T_c_, where T_c_ corresponds to the mean waiting time measured in a given area), (3) mean jump lengths, (4) standard deviation of jump lengths (σ), and (5) D_b_ (particle tracking biodiffusion coefficient). As pointed out by Bernard *et al*. [36], the detection of a jump in a given area depends not only on particle mixing intensity but also on the density of luminophores in this area. The computation of the normalized number of jumps therefore provides a measure of the probability of jump and therefore of particle mixing intensity. Inversed waiting time was used instead of waiting time because it also measures the frequency of mixing events and co-varies positively with the normalized number of jumps. D_b_ was computed based on Meysman *et al*. [29] using the following formula:

$$D_{b}=\sigma^{2}/\left(4T_{c}\right)$$

Where D_b_ is the particle tracking biodiffusion (in cm².yr^-1^) coefficient, σ the standard deviation of jump lengths (in cm) and T_c_ the average waiting time (in years).

For each experiment, all profiles were computed for 5 experiment durations (i.e., 6, 12, 24, 36 and 48 hours).

All the above-described procedures were carried out using specific routines developed with the R free software language and environment for statistical computing (R Core Team, 2013. R foundation for statistical computing, Vienna, Austria. URL: http://www.R-project.org/).

### 2.5. Data analysis

The effects of tested factors (see below) on the location of the vertical profiles of all the above-mentioned parameters were assessed using PERmutational Multivrariate ANalyses Of VAriances (PERMANOVAs) [44, 45] without preliminary data transformation. We used the Euclidean distance to assess dissimilarities between profiles. Our overall design consisted in 3 fixed factors, namely *Temperature* (*Te*, 2 levels), *Food addition* (*Fo*, 2 levels), and *Experiment duration* (*Ed*, 5 levels) together with a fourth random factor “Replicates” (*Rep*), which was nested within *Se* and *Fo*. In case of significant interactions between factors, pairwise tests were performed to characterize their modalities. The effects of the tested factors on the dispersion (i.e., among experiment variability) of vertical profiles were checked using the Permdisp procedure [46] (same distance and same design as described above).

All the above-described procedures were performed using the PRIMER® v6 package with the PERMANOVA+ add-on software [47, 48].

## Results

### 3.1. Main effects

Table 1 shows the results of the PERMANOVA and the PERMDISP procedures carried out on all vertical profiles.

**Table 1:** Results from PERMANOVA and PERMDISP analyses for differences in vertical depth profiles of normalized number of jumps (jumps.luminophore^-1^), inversed waiting times (h^-1^), jump characteristics (means and standard deviations of jumps lengths in mm) and Db (cm^2^.yr^-1^), amongst *Temperature* (*Te*), *Food addition* (*Fo*) and *Experimental duration* (*Ed*), based on a Euclidean resemblance matrix.

*Factors*		*Normalized number of jumps*	*Inversed waiting times*	*Mean jump lengths*	*σ of jump lengths*	*Db*
*Te*	df	1	1	1	1	1
	MS	0.6	411.82	0.49	0.67	61.73
	Pseudo-F	7.06*	3.96	5.03	3.91*	1.99
	p(perm)	0.0013	0.0054	0.0044	0.0112	0.0276
*Fo*	df	1	1	1	1	1
	MS	1.88E-2	152.79	0.26	0.55	42.66
	Pseudo-F	0.22	1.47	2.68*	3.2*	1.38
	p(perm)	0.7975	0.1673	0.0451	0.0214	0.1569
*Ed*	df	4	4	4	4	4
	MS	1.2E-2	325.52	3.8E-2	5.78E-2	74.96
	Pseudo-F	1.23	7.8*	3.32	2.59	4.68*
	p(perm)	0.2751	0.0001	0.0003	0.0003	0.0001
*TexFo*	df	1	1	1	1	1
	MS	2.06E-2	51.71	0.16	0.23	27.19
	Pseudo-F	0.24*	0.5	1.63*	1.31*	0.88
	p(perm)	0.7594	0.8437	0.1663	0.2405	0.557
*TexEd*	df	4	4	4	4	4
	MS	7.71E-3	38.12	9.97E-3	1.79E-2	11.45
	Pseudo-F	0.79*	0.91*	0.87	0.8	0.72*
	p(perm)	0.6027	0.5543	0.6017	0.6942	0.9367
*FoxEd*	df	4	4	4	4	4
	MS	3.73E-3	84.76	9.83E-3	2.63E-2	28.77
	Pseudo-F	3.8	2.03*	0.86*	1.18	1.8*
	p(perm)	0.0006	0.0063	0.6144	0.2517	0.0036
*Rep*	df	28	28	28	28	28
	MS	8.54E-2	104.07	9.7E-2	0.17	30.88
	Pseudo-F	8.71*	2.49	8.47*	7.75*	1.93
	p(perm)	0.0001	0.0001	0.0001	0.0001	0.0001
*SexFoxEd*	df	4	4	4	4	4
	MS	2.52E-2	22.54	9.92E-3	2.21E-2	13.34
	Pseudo-F	2.57*	0.54*	0.87*	0.99	0.83*
	p(perm)	0.0144	0.9814	0.5994	0.4484	0.7741

*: PERMDISP, p<0.05

*Te* significantly affected the location of the vertical profiles for all tested parameters together with their dispersion for normalized number of jumps and σ of jump lengths. Conversely, *Fo* only affected the location and dispersion of the vertical profiles of jump characteristics. *Ed* also significantly affected the location of the vertical profiles for all tested parameters but normalized numbers of jumps. There were no interactions between the effects of *Te* and *Fo* and between those of *Te* and *Ed*. Conversely, there was a significant interaction between the effects of *Fo* and *Ed* on the location of the vertical profiles of: (1) normalized numbers of jumps, (2) inversed waiting times, and (3) D_b_. *Rep* significantly affected the location of the vertical profiles for all tested parameters. The interactions between the different levels of *Te*, *Fo* and *Ed* only affected the location and dispersion of the vertical profiles of the normalized numbers of jumps.

### 3.2. Normalized numbers of jumps

**Fig 3:**
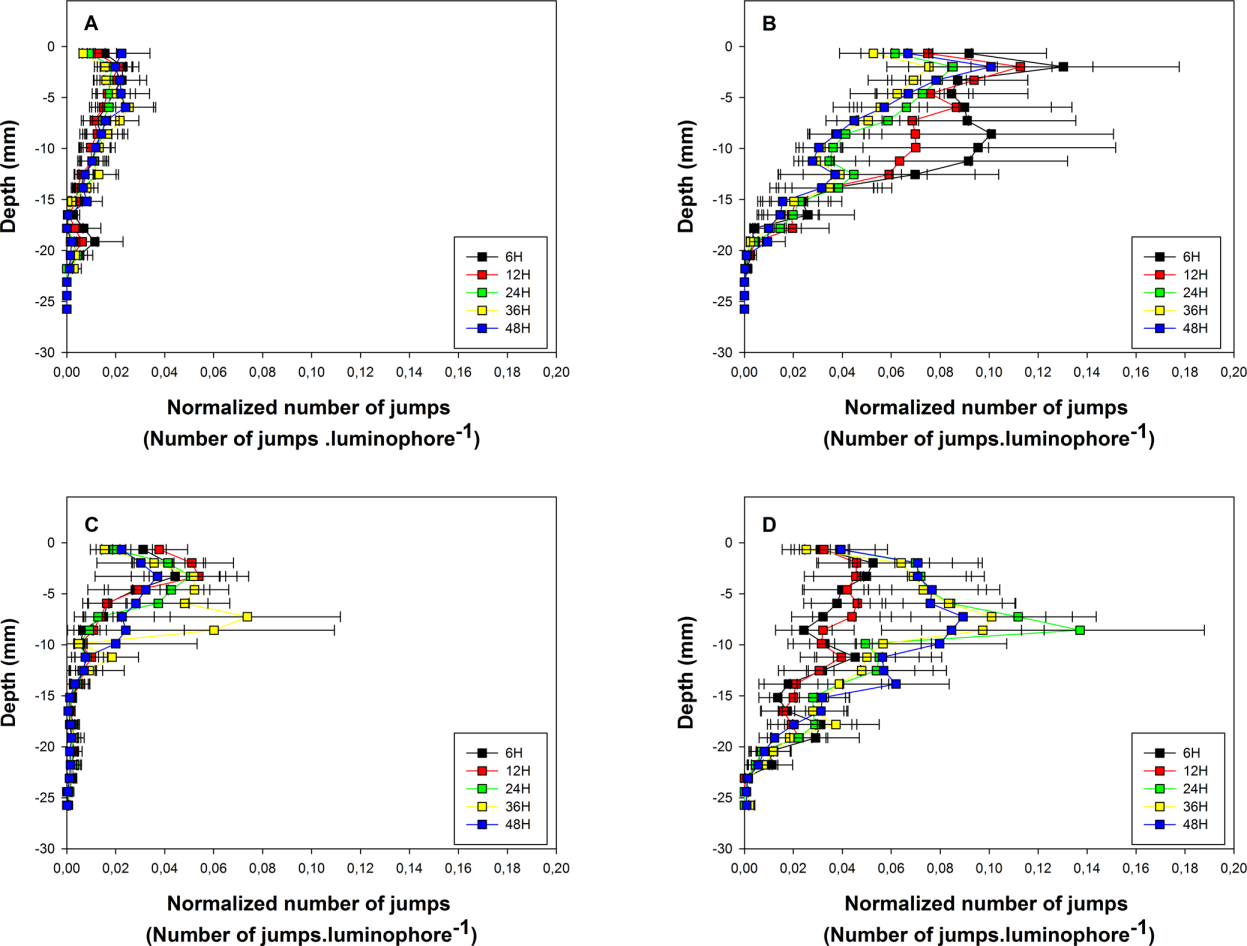
Vertical profiles of the normalized number of jumps. Vertical profiles were recorded during the: autumn without food addition experiments (A), summer without food addition experiments (B), autumn with food addition experiments (C) and summer with food addition experiments (D). Colors correspond to different experiment durations. Horizontal bars are standard errors and refer to between-experiment variability.

The vertical profiles of normalized number of jumps are shown in Fig 3 for all combinations of the different levels of *Te*, *Fo* and *Ed*.

During the autumn temperature experiments without food addition (Fig 3A), average normalized numbers of jumps were low (less than 0.02 jumps.luminophore^-1^ recorded for the total duration of the experiment) and variability was limited among experiments. Average normalized numbers of jumps were maximal immediately below the sediment-water interface and then decreased with depth in the sediment column. There were no effects of *Ed* on the location and the dispersion of the vertical profiles of normalized numbers of jumps (Table 1).

Average normalized numbers of jumps were higher during the summer temperature experiments without food addition (Fig 3B) than during the autumn temperature experiments without food addition with a maximal value of 0.13 jumps.luminophore^-1^ (Tables 1 and 2).

**Table 2:** Results of pairwise PERMANOVA and PERMDISP analyses for differences in vertical depth profiles of normalized number of jumps and inversed waiting time among levels of *Te* within all possible combinations of *Fo* and *Ed*, based on Euclidean resemblance matrix.

		*Normalized number of jumps*		*Inversed Waiting time*	
		No food	Food	No food	Food
		Autumn *vs* Summer	Autumn *vs* Summer	Autumn *vs* Summer	Autumn *vs* Summer
6H	t	1.70*	1.49	1.04	0.92
	p	**0.0322**	0.054	0.3431	0.4612
12H	t	1.89*	1.42	**1.50**	1.08
	p	**0.004**	0.1146	**0.0283**	0.249
24H	t	1.89*	2.11*	**1.73**	1.14
	p	**0.0161**	**0.0107**	**0.0327**	0.1775
36H	t	1.63	1.17	1.09	1.22
	p	0.0578	0.219	0.2912	0.0712
48H	t	**1.67**	2.02*	1.74*	1.09
	p	**0.0337**	**0.0286**	**0.0113**	0.2676

*: PERMDISP, p<0.05

Among experiments variability was also higher during the 6, 12 and 24 h experiments (Table 2). There were no significant effects of *Ed* on the vertical profile of normalized numbers of jumps (Table 3) and the shapes of these profiles were similar to those recorded during the autumn temperature experiments without food addition. This trend was especially marked for the 24, 36 and 48 h experiments.

**Table 3:** Results of pairwise PERMANOVA and PERMDISP analyses for differences in vertical depth profiles of normalized number of jumps and inversed waiting time among levels of *Ed* within all possible combinations of *Te* and *Fo*, based on Euclidean resemblance matrix.

		*Normalized number of jumps*				*Inversed Waiting time*			
		Summer		Autumn		Summer		Autumn	
		No food	Food	No food	Food	No food	Food	No food	Food
6H *vs* 12H	t	1.04	1.02	0.82	0.69	1.15	**1.86**	**1.59**	1.04
	p	0.3623	0.41	0.6688	0.625	0.2	**0.0439**	0.0421*	0.3579
6H *vs* 24H	t	1.26	**2.08**	0.78	0.93	**1.79**	**1.98**	**2.17**	1.10
	p	0.2394	**0.0099**	0.6495	0.4319	0.0051*	0.0105*	0.0003*	0.2329
6H *vs* 36H	t	1.26	**2.27**	0.87	1.25	**2.06**	**1.66**	**2.20**	0.96
	p	0.2456	**0.0144**	0.5262	0.174	0.0013*	**0.0185**	0.0004*	0.5198*
6H *vs* 48H	t	1.31	**1.94**	0.65	1.19	**2.17**	**1.64**	**2.37**	1.14
	p	0.2387	**0.048**	0.86	0.2236	0.0005*	**0.012**	0.0004*	0.1626*
12H *vs* 24H	t	1.26	**2.14**	1.0513	0.95	**1.65**	1.38	**2.48**	1.23
	p	0.2569	**0.0145**	0.323	0.4114	**0.0144**	0.1514	**0.0001**	0.2969
12H *vs* 36H	t	1.24	**2.56**	1.06	1.22	**2.18**	1.0802	**2.50**	0.96
	p	0.2565	**0.0079**	0.3206	0.1925	**0.0002**	0.2886	**0.0001**	0.5602
12H *vs* 48H	t	1.32	**2.11**	0.72	1.17	**2.13**	1.11	**3.04**	1.14
	p	0.2183	**0.0395**	0.7594	0.2381	0.0002*	0.2564	0.0001*	0.2056*
24H *vs* 36H	t	0.94	0.87	1.01	1.19	**2.47**	0.9	1.38	0.73
	p	0.466	0.5118	0.3792	0.187	**0.0008**	0.6762	0.2439	0.7917
24H *vs* 48H	t	1.03	0.97	0.76	0.95	**2.46**	0.93	**1.39**	0.84
	p	0.409	0.3995	0.6937	0.4249	**0.001**	0.6235	0.0025*	0.7052
36H *vs* 48H	t	0.88	1.03	1.13	1.0894	1.38	0.79	1.08	1.4647
	p	0.5066	0.3411	0.2935	0.3094	0.2125	0.5378	0.1797	0.0577

*: PERMDISP, p<0.05

The average normalized numbers of jumps in the upper part of the sediment column seemed higher during the autumn temperature experiments with food addition (Fig 3C) than during the autumn temperature experiments without food addition with a maximal value of 0.07 jumps.luminophore^-1^. However, there were no significant effects of *Fo* on the location of whole vertical profiles (Tables 1 and 4). There were no significant effects of *Ed* on the location of vertical profiles as well (Table 3) and the shapes of these profiles were similar to those recorded during the autumn temperature experiments without food addition except for the 36 h experiments, which showed a marked subsurface (at ca. 7 mm deep) maximum.

**Table 4:** Results of pairwise PERMANOVA and PERMDISP analyses for differences in vertical depth profiles of normalized number of jumps and inversed waiting time among levels of Fo within all possible combinations of Te and Ed, based on Euclidean resemblance matrix.

		*Normalized number of jumps*		*Inversed Waiting time*	
		Autumn	Summer	Autumn	Summer
		No food *vs* Food	No food *vs* Food	No food *vs* Food	No food *vs* Food
6H	t	0.60	1.19	0.99	1.25
	p	0.8542	0.2631	0.4471	0.1494
12H	t	0.59	0.92	0.89	1.12
	p	0.8302	0.4214	0.7192	0.2556
24H	t	1.05	1.11	0.80	1.09
	p	0.3589	0.296	0.9989	0.2808
36H	t	1.12	1.02	1.04	0.83
	p	0.2734	0.3527	0.3464	0.8778
48H	t	0.65	1.11	1.04*	0.96
	p	0.8131	0.2942	0.3621	0.5507

*: PERMDISP, p<0.05

*Ed* significantly affected the location (but not the dispersion) of the vertical profiles of normalized numbers of jumps during the summer temperature experiments with food addition (Fig 3D, Table 3). Vertical profiles recorded during the 6 and 12 h experiments did not differ between one another but did significantly differ from those recorded during the 24, 36 and 48 h experiments. Corresponding maximal values were 0.05 and 0.14 jumps.luminophore^-1^ for shorter and longer experiments, respectively. For the 6 and 12 h experiments, there were no significant differences in the location of the vertical profiles recorded during the summer and the autumn temperature experiments with food addition (Fig 3C and 3D, Table 2). Conversely, there were significant differences in the location of the corresponding vertical profiles recorded during the 24 and 48 h experiments. In this last case, normalized numbers of jumps were higher during summer (maximal value of 0.14 jumps.luminophore^-1^) than during autumn temperature experiments (maximal value of 0.07 jumps.luminophore^-1^). The vertical profiles recorded during the 24, 36 and 48 h of summer temperature experiments with food addition were all characterized by a subsurface (at ca. 8 mm deep) maximum. This was rather different from the patterns observed during the summer temperature experiments without food addition. However it should be underlined that, for all experiment durations, there were no statistically significant differences in the location of the whole vertical profiles between the summer temperature experiments with and without food addition (Fig 3B and 3D, Table 4).

### 3.3. Inversed waiting times

**Fig 4:**
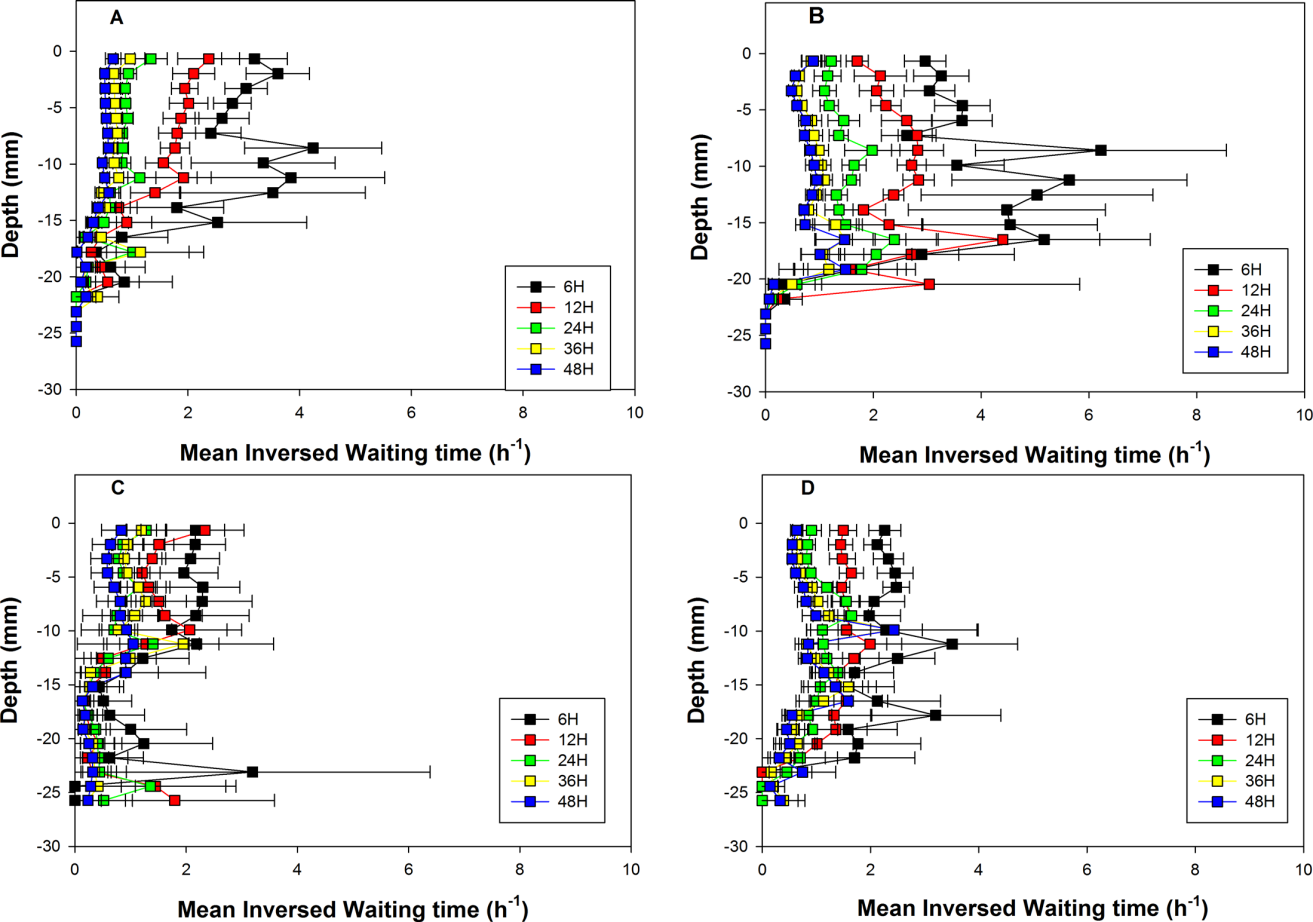
Vertical profiles of inversed waiting times. Vertical profiles were recorded during the: autumn without food addition experiments (A), summer without food experiments (B), autumn with food addition experiments (C) and summer with food addition experiments (D). Colors correspond to different experiment durations. Horizontal bars are standard errors and refer to between-experiment variability.

The vertical distributions of the average inversed waiting times are shown in Fig 4 for all combinations of the different levels of *Te*, *Fo* and *Ed*. The effect of *Te* on the location of vertical profiles was significant and similar to the one found for normalized numbers of jumps with a trend toward higher values during summer than during autumn temperature experiments without food addition. The effect of *Ed* on the location of vertical profiles was significant for all combinations of *Te* and *Fo* but the autumn temperature experiments with food addition (Table 3).

Whenever significant, the effect of *Ed* corresponded to a decrease in inversed waiting times with experiment duration (Table 3). *Fo* interacted significantly with *Ed* in affecting the location of vertical profiles (Table 1) by reducing: (1) the overall range of inversed waiting times (maximal values of inversed waiting times of 3.5 vs. 5.6 h^-1^ during summer temperature experiments with and without food addition, respectively), and (2) the experiment duration required to reach a stable vertical profile (eg, 12 vs. 36 h during summer temperature experiments with and without food addition, respectively).

### 3.4. Jump characteristics

The location of the vertical profiles of jump length and σ were both significantly affected by *Te*, *Fo* and *Ed* without any significant interactions between any of them (Table 1).

**Fig 5:**
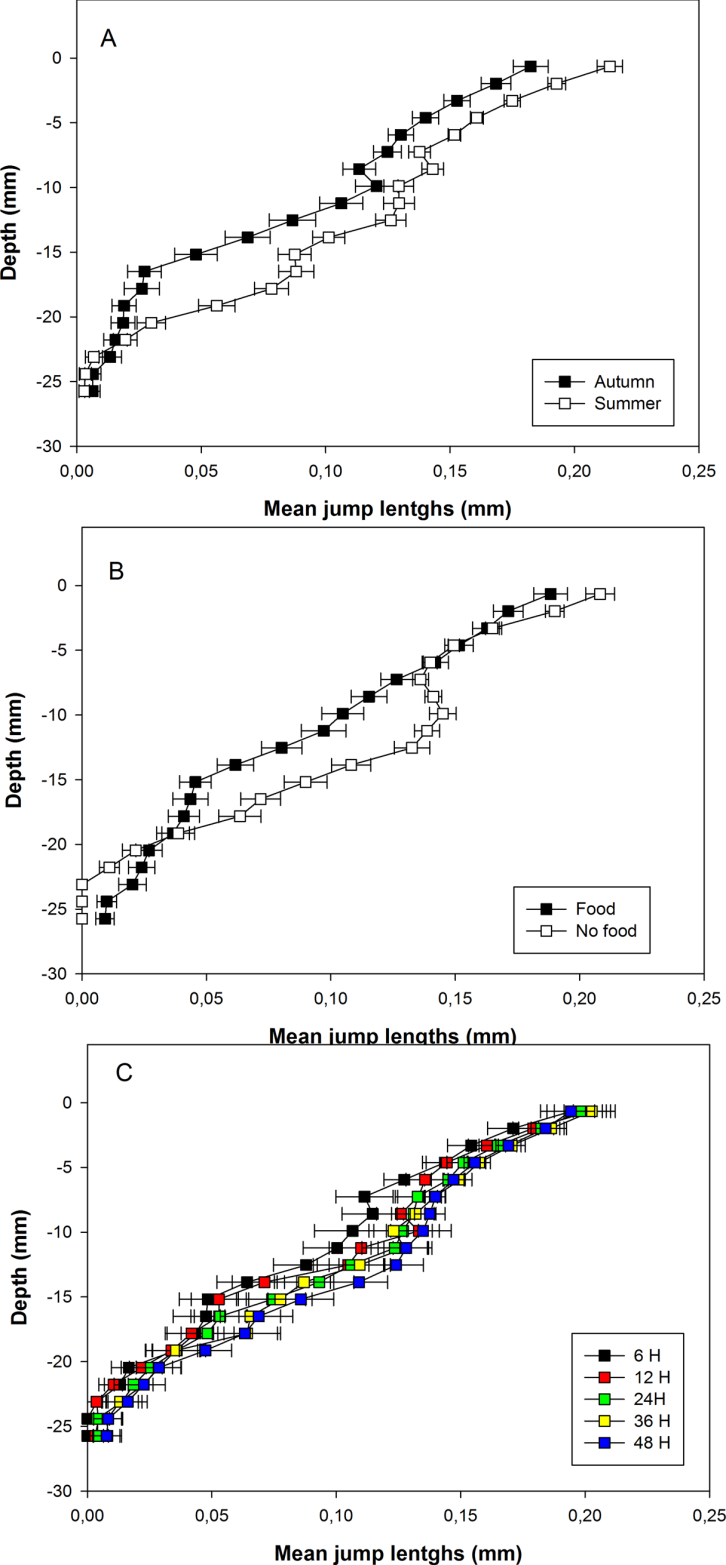
Vertical profiles of mean jump lengths. Vertical profiles are shown for pooled autumn and summer temperature experiments (A), for pooled with and without food addition experiment (B), and for all pooled experiments of the same duration (C). Colors correspond to different experiment durations. Horizontal bars are standard errors and refer to between-experiment variability.

Average jump lengths tended to decrease with depth (Fig 5) and were significantly higher during summer than during autumn temperature experiments (Fig 5A). There was no effect of *Fo* on mean jump length close (i.e., within the 7 mm top sediment) to the sediment water-interface. Average jump lengths were then higher without food addition from 7 to 19 mm deep in the sediment (Fig 5B). Average jump lengths also tended to increase with experiment duration.

**Fig 6:**
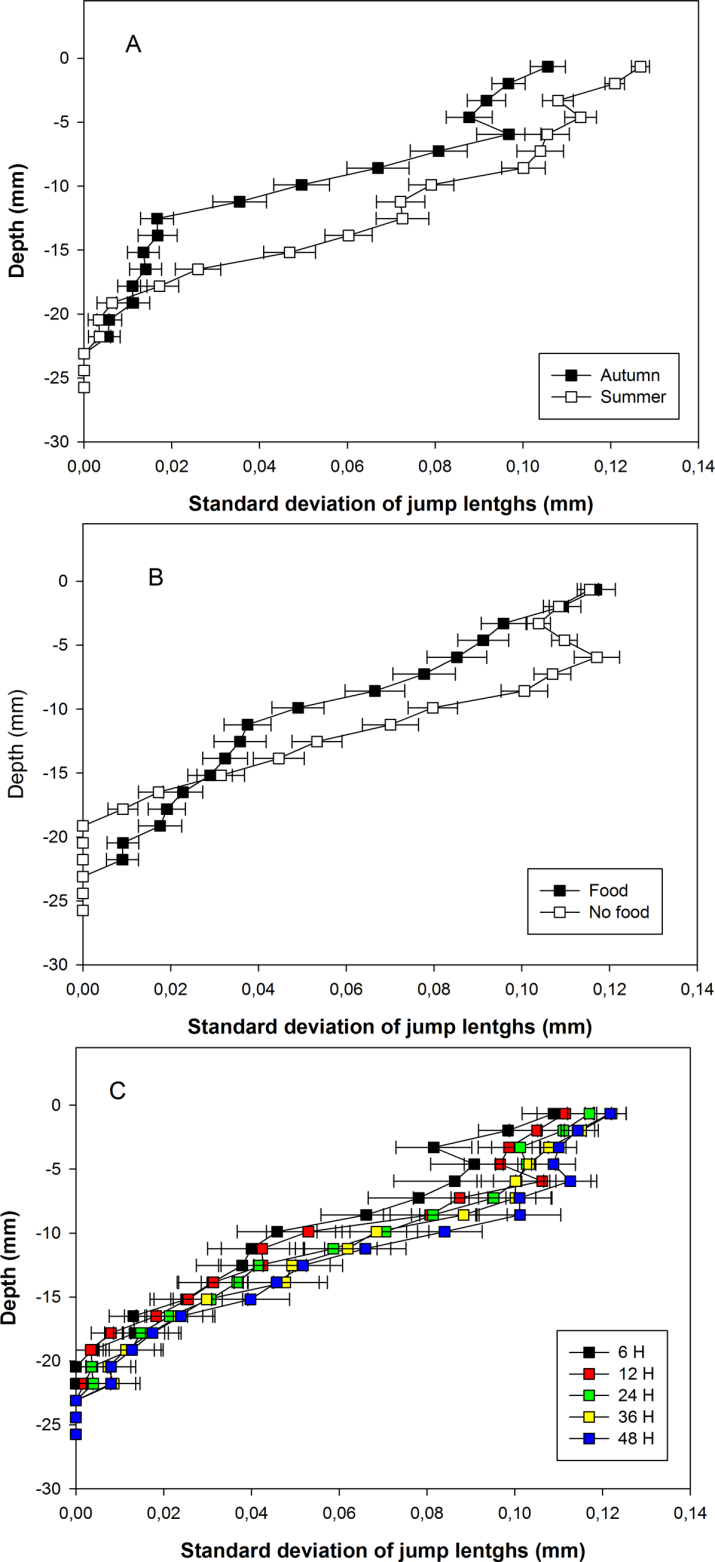
Vertical profiles of standard deviations of jump lengths. Vertical profiles are shown for pooled autumn and summer temperature experiments (A), for pooled with and without food addition experiments (B), and for all pooled experiments of the same duration (C). Colors correspond to different experiment durations. Horizontal bars are standard errors and refer to between-experiment variability.

σ followed the same general pattern with: (1) a diminution with depth in the sediment column, (2) higher values without food addition, and (3) a general increase with experiment duration. There was only a minor difference regarding the range of depths (i.e., from 3 to 15 mm) where standard deviations were higher without than with food addition) (Fig 6).

### 3.5. D_b_

The locations of the vertical profiles of D_b_ were significantly affected by *Te* and *Ed*. In addition, there was a significant interaction between *Ed* and *Fo* (Table 1).

**Fig 7:**
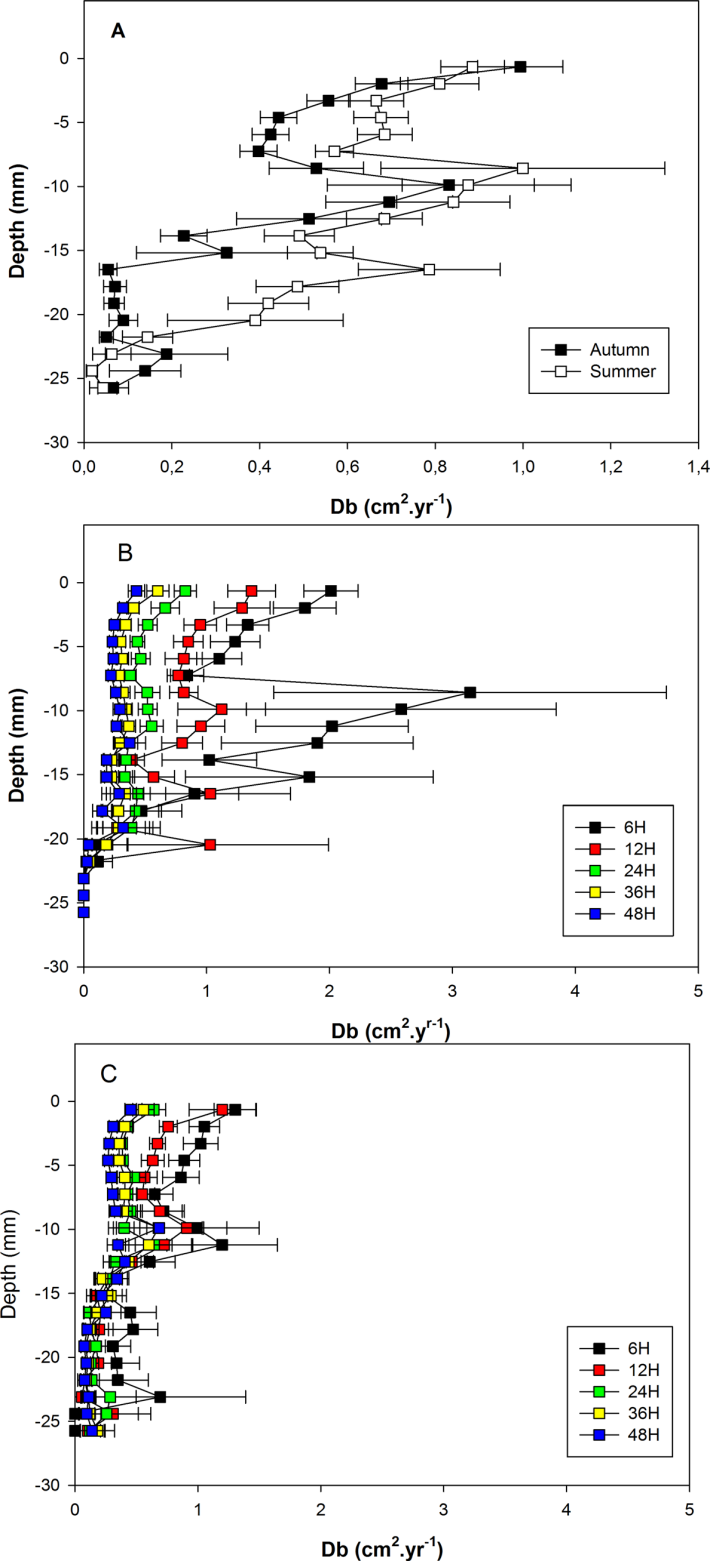
Vertical profiles of D_b_. Vertical profiles are shown for pooled autumn and summer experiments (A), for pooled experiments of the same duration without food addition (B), and for pooled experiments of the same duration with food addition. Horizontal bars are standard errors and refer to between-experiment variability.

Overall, D_b_ were higher during summer than during autumn temperature experiments (Fig 7A). For both temperature treatments, there were two peaks in vertical profiles: a first one close to the sediment-water interface and a second one between 8 and 11 mm deep in the sediment column. During summer temperature experiments, there was also a third peak located between 16 and 17 mm deep in the sediment.

The vertical profiles of D_b_ are shown in Fig 7B and 7C for all combinations of *Ed* and *Fo*. In both food conditions, the effect of *Ed* was associated with a trend toward decreasing D_b_ with increasing experiment duration. As observed for inversed waiting times, this effect was less pronounced when food was added, due to: (1) a reduction in the overall range of inversed waiting times (eg, maximal values of mean inversed waiting times of 1.30 vs. 3.14 h^-1^ during experiments with and without food addition, respectively), and (2) a reduction in the experiment duration required to reach a stable vertical profile (eg, 24 and 36 h during experiments with and without food addition, respectively). Only during the shortest experiment duration (i.e. 6 h), vertical profiles exhibited significantly higher D_b_ without than with food addition (Fig 7B and 7C).

## Discussion

### 4.1. Methodological considerations when assessing environmental effects on particle mixing process using direct measurements of particle mixing fingerprints

Our results were obtained based on measurements of individual luminophore displacements [36]. The present study constitutes the first attempt to assess the effect of environmental factors on particle mixing fingerprints using this approach. This requires several methodological considerations regarding: (1) the appropriate descriptor of jump frequency and (2) the possibility to measure and compare vertical profiles instead of overall mean values of each considered parameter.

#### 4.1.1. Descriptor of jump frequency

In the CTRW model, particle displacements are controlled by two main types of parameters: the frequency of jumps and the characteristics (including their length) of individual jumps [29, 30, 31]. The frequency of jumps is usually described through the frequency distributions of waiting times, which are defined as the time intervals between two consecutive jumps of the same individual particle [29, 30, 32]. Our results clearly show that waiting times are affected by experimental duration, which simply results from the fact that it is not possible to measure a waiting time longer than experimental duration. The frequency of jumps can however also be approached through the assessment of the probability of jumps of a luminophore during an elementary time interval. During the present study, this was achieved through the computation of the normalized number of jumps (i.e., the proportion of the total number of luminophores that have jumped within an elementary time interval). This parameter is closely related to the “activity” parameter proposed by Schiffers *et al*. [49] with the difference that the normalized number of jumps is based on the assessment of individual luminophore displacements and not on changes in luminophore concentrations. During the present study, the normalized number of jumps was integrated over time periods of increasing durations to become more comparable with waiting times. Our results show that the normalized number of jumps was not affected by experiment duration. They therefore suggest that it constitutes a better proxy of jump frequency than waiting time when assessing particle mixing fingerprints based on the measure of individual particle displacements.

#### 4.1.2. Use of vertical profiles

The experimental approach used during the present study presents some advantages relative to the classical assessment of particle mixing through the coupling of the experimental assessment of luminophore vertical profiles and modeling. This last approach indeed supposes spatially homogeneous particle mixing and produces: (1) single distributions of waiting times and jump lengths and (2) a single value of D_b_ for each experiment (i.e., combination of *Te*, *Fo* and *Ed* in the case of the present study). Conversely, the use of our approach results in a 2D description of particle mixing [36], which presents a higher degree of finesse in describing particle mixing and thus a higher potential in assessing the effect of environmental factors on particle mixing. According to Bernard *et al*. [36], the horizontal component of particle mixing is highly variable among clams because mostly controlled by the geometry of the siphonal gallery network and resulting from processes occurring over short time scales such as the preferential use of a siphonal gallery during the duration of the experiment. There is thus no real sense in considering explicitly this component when assessing the effects of environmental factors on particle mixing. These authors also showed that, in *A*. *alba*, particle mixing fingerprints varied with depth in the sediment column. This pattern is more constant between individuals because corresponding to changes in different types of activities occurring at different depths due to the morphology of the clams and of their positioning within the sediment column. The comparison of vertical profiles of the values of the different parameters characterizing particle mixing may therefore prove both more realistic and more discriminative (i.e., between the different levels of tested environmental factors) than the crude comparison of their mean values.

### 4.2. Temperature associated changes in particle mixing fingerprints

The comparisons of vertical profiles clearly show that particle mixing fingerprints in *A*. *alba* differ between the two tested temperature conditions. Particle mixing was stronger at summer than at autumn temperature. All tested parameters were significantly affected by *Te* (Table 1) and the analysis of the corresponding profiles shows that these parameters were affected all over the sediment column. Temperature associated differences thus affected the three vertical functional areas identified by Bernard *et al*. [36], namely: the sediment-water interface, the network of siphonal galleries and to a lower extent the shell area. Both proxies of jump frequency tended to be higher at summer than at autumn temperature all over the sediment column (Figs 1 and 2). Jump lengths were also longer and more variable during summer than during autumn temperature experiments (Figs 3A and 4A). Consequently D_b_ were higher during summer than during autumn temperature experiments (Fig 5A). In this last case, differences were almost null close to the sediment-water interface and maximal deep in the sediment column (i.e., within the shell area).

To our knowledge, only one study based on the CTRW model has been carried out to assess the effect of temperature condition on particle mixing by *A*. *alba* [33]. These authors reported no significant differences in the proportions of luminophores found deeper than 0.5cm and in D_b_ between summer and winter. Conversely, we observed significant differences in particle mixing intensity (including D_b_). Braeckman *et al*. [33] used a classical CTRW approach and therefore derived D_b_ from the fitting of luminophore profiles using a CTRW model. Therefore, they only had access to mean values of D_b_, whereas our approach was based on the assessment of individual jumps and therefore allowed for the comparisons of profiles (see above).

Conversely, in the closely related bivalve *A*. *ovata* Maire *et al*. [17] reported, using a classical CTRW modeling approach, lower waiting times and more variable jump lengths (higher σ) in summer than in autumn, and consequently, higher D_b_ in summer than in autumn. From a qualitative standpoint, these results are similar to ours. Nevertheless, our results also show that mean jump lengths and not only their σ were higher for summer than for autumn temperature. In the genus *Abra*, particle mixing is clearly resulting from clam’s activity [18]. Grémare *et al*. [13] studied the effect of temperature on the intensity of siphonal activity in *A*. *nitida*. They use two independent indices describing this activity, namely the percentage of time active (ACT) and the mean activity per time active (MATA). In both cases they reported a significant and positive effect of temperature. ACT is indicative of the frequency of feeding and thus indirectly of the frequency of jumps induced by this activity. In this sense, differences in both indices of frequency of jumps recorded during the present study could result from the effect of temperature on clam’s activity. MATA is indicative of the surface prospected by the inhalant siphon during feeding and is therefore indicative of the extension of this siphon (*i*.*e*. the higher the MATA, the more extended the siphon). A positive effect of temperature on MATA (as in [13]) could therefore account for the occurrence of longer and more variable jumps at the sediment-water interface during the present study. The occurrence of longer and more variable jumps within the sediment column could result as well from differences in the patterns of siphonal activity induced by temperature. Deposit-feeding bivalves are known to destabilize the sediment between the mounds they create [50], *i*.*e*. in areas corresponding to the network of siphonal galleries. In this sense, the higher siphonal activity found at summer temperature could explain the occurrence of long and variable jumps in breaking the elastic sediment matrix and therefore making particles easier to displace over longer distances. Such an impact of benthic organisms on the visco-elastic properties of sediment has already been demonstrated for burrowing worms [51].

### 4.3. Effect of food availability on particle mixing fingerprints

#### 4.3.1. Effect on jump frequency

The vertical profiles of the normalized number of jumps were significantly affected by the interactions between *Te*, *Ed* and *Fo*. During autumn temperature experiments, and for all experimental durations, vertical profiles were not significantly affected by *Fo*. During summer temperature and without food addition experiments, vertical profiles were not significantly affected by *Ed*. Conversely, with food addition, vertical profiles differed significantly for short (i.e., 6 and 12h) and long (i.e., 24, 36 and 48h) experiments. In this last case, differences between profiles mostly resulted from the occurrence of lower normalized numbers of jumps in the upper part of the sediment column when food was added.

A similar negative effect has been reported by Maire *et al*. [16] in *A*. *ovata*. These authors tested 3 levels of addition of phytoplanktonic detritus and reported a decrease in D_b_ at the highest concentration. Their results supported those of Grémare *et al*. [13], who previously reported a decrease in the the siphonal activity of the same species at high food concentration. Both authors reported that the functional responses of the closely related species *A*. *nitida* was completely different and characterized by a constant increase in siphonal activity and particle mixing activities with increasing food concentrations. Their interpretation was that the individuals of *A*. *nitida* originating from Swedish waters were more adapted to strong and restricted in time phytoplanktonic blooms [52] than the individuals of *A*. *ovata* originating from a Mediterranean lagoon. The individuals of *A*. *alba* used during the present study were collected in the Arcachon Bay, where seasonal changes (i.e., in terms of timing and intensity) in primary production are relatively close to those of the Mediterranean Sea [53, 54]. Our hypothesis is thus that, during our experiments, food addition has induced an inhibition of particle mixing in *A*. *alba* as well. The fact that the inhibition recorded during the present study was restricted to short experimental durations is probably linked to a progressive impoverishment of the sediment-water interface following a punctual addition of phytoplankton detritus as already suggested by Maire *et al*. [16].

Besides changes in the intensity of deposit-feeding, switch in feeding behavior may however also contribute to account for the transitory inhibition of particle mixing. According to Levinton [55] *“there appears to be no exclusively deposit-feeding Tellinacea”*. Moreover, several species of the genus *Abra*, including *A*. *alba*, have been shown to switch between deposit and suspension feeding [56]. In this last case, the inhalant siphon remains immobile in the water column for long periods of time [13] and then sediment particle movements resulting from siphonal become scarcer and also shorter. Together with local hydrodynamics [55, 57], which were constant during our experiments, concentration of suspended particulate organic matter is known to constitute one of the main controlling factors of the switch between deposit and suspension feeding [58, 59]. Grémare *et al*. [13] also observed suspension-feeding in *A*. *nitida* mostly immediately after food addition. Our results suggest that this is also the case in *A*. *alba* since visual inspections of our video records showed that suspension feeding did occur in the beginning (i.e. within the first hour) of 10 out of our 16 with food addition experiments versus only 1 out of 16 of our without food experiments (GB, personal observations). The transitory inhibition of particle mixing by *A*. *alba* may thus partly result from a switch in feeding mode and not only from a decline in deposit-feeding intensity as already suggested for *A*. *ovata* [16]. This inhibition was not observed in autumn temperature, which could be related to the fact that, under these temperature conditions, feeding activity is severely limited by low temperature (see above, [17]).

#### 4.3.2. Effect on jump characteristics

Vertical profiles of both jump characteristics (i.e., mean jump lengths and σ) were significantly affected by *Fo* without any significant interaction with the two other tested factors (Table 1). This effect consisted in shorter mean jump lengths and lower σ within the depth range of the sediment column corresponding to the network of siphonal galleries when food was added. Vertical profiles of these two parameters showed regular decreases with depth when food was added. Conversely, without food addition, those vertical profiles exhibited a subsurface peak located within the depth range corresponding to the network of siphonal galleries. The difference in these two patterns suggests that foraging behavior by the siphons below the sediment-water interface differed without and with food addition. Here again, these results are indicative of an inhibition of particle mixing by food addition. Two hypotheses may be raised to explain them. As explained above, food addition may at certain concentration inhibit siphonal activity at the sediment-water interface and thus particle mixing including jump characteristics in individuals of the genus *Abra* originating from temperate populations (see above) as discussed above for jump frequency. However, this hypothesis does not appear fully satisfactory because, the upper part of the profiles of both jump lengths and σ were not affected by food addition. Maire *et al*. [18] reported that: (1) siphonal activity of *A*. *ovata* tends to decrease with experiment duration, and (2) *A*. *ovata* never explores the same subarea of the sediment surface before the total area delimited by the extension of their inhalant siphon has been fully prospected. This suggests that the impoverishment of the surface sediment in organic matter constitutes a key factor in controlling the feeding activity in *A*. *ovata*. During our experiments, such an impoverishment was probably more pronounced without food addition due to: (1) lower initial concentration during the without food addition experiments, and (2) the transitory inhibition of deposit-feeding during the with food addition experiments (see above). Moreover, Amouroux *et al*. [60] observed a regular current induced by the exhalent siphon of *A*. *ovata* in the section of their burrow where faeces are stored and suggested that this was indicative of a “gardening” behavior. During our experiments, longer and more variable jumps below the sediment-water interface without food addition could therefore have been caused by movements of the inhalant siphon foraging on gardened faeces in response to an impoverishment of the sediment-water interface during the curse of our experiments.

#### 4.3.3. Effect on D_b_

Together with σ, mean waiting times are one of the two components involved in the computation of D_b_ [29]. During the present study, changes in vertical profiles of D_b_ were highly similar to those of inversed waiting times. They also showed a transitory inhibition following food addition during summer temperature experiments as already shown by Maire *et al*. [16] at high food concentration for individuals of *A*. *ovata* originating from a temperate population. Overall D_b_ thus appeared to be more controlled by T_c_ than by σ. Such a dependency explains why D_b_ were also significantly affected by *Ed*, which contributes to complicate the assessment of the effects of environmental parameters during long term experiments by buffering D_b_ values. This problem could be handled by deriving estimates of T_c_ (and thus D_b_) from another proxy of jump frequency, which would be independent of *Ed*. Our results suggest that this proxy could be the normalized number of jumps.

## Conclusions and Perspectives

The use of a new approach allowing for the tracking of individual particles at a high temporal resolution proved efficient in assessing the effects of *Te* and *Fo* on particle mixing in the deposit-feeding bivalve *A*. *alba*. Our main conclusions regarding these effects are as follows:

1. Sediment particle displacements were longer, more variable and more frequent during experiments carried out at summer temperature. This is coherent with the fact that temperature has been shown to positively affect both siphonal activity and particle mixing in closely related species. Conversely, limitations by low temperature precluded any significant effect of food addition at autumn temperature experiments.
2. At summer temperature, food addition induced a transitory inhibition of particle mixing, which resulted from both: (1) a functional response of deposit-feeding to food addition (as already described in a closely related species at high food concentrations), and (2) a switch from deposit- to suspension-feeding immediately following food addition.

From a methodological standpoint, our results show that the estimates of average waiting time (generally used to assess the jump frequency of particle when describing sediment particle mixing process with CTRW model) and thus D_b_ are strongly *Ed* dependent. In this sense, the present study highlights the need for a better descriptor of jump frequency at short time scale during experiments based on the tracking of individual particles. We propose to use the normalized number of jumps for this purpose because: (1) it was not significantly affected by experiment duration, and (2) it allowed for the detection of the negative effect of food addition on the frequency of jump during summer temperature experiments, which was not the case of waiting time. A challenge for future research thus consists in deriving a proxy of waiting times independent of experiment duration from this parameter. In any case, future studies assessing on the effects of environmental variables on sediment particle mixing process should carefully pay attention to observational time and spatial scales to capture changes in benthic fauna behavior and their consequences, which can be restricted both in time and to specific areas of the sediment column.

## Supporting Information

S1 Software Compiled version of the image analysis software used (TracklumS, technical requirements, installation and execution instructions can be found in the pdf- or txt-file “ReadMe”), together with a time-lapse sequence of 500 original images (abra_exp3.avi that can be found in the “DemoFilm” folder), a guide on how to use and parametrize this code to visualize data and to reproduce the raw data mentioned below (pdf-file HowTo), and (4) raw data regarding isolated luminophores, waiting times and jump lengths isolated from the above-mentioned time-lapse sequence (in txt format that can be found in the DemoFilm folder, see the pdf-file “HowTo”). (DOCX)
