# Supplementary material for: Experimental Assessment of the Effects of Temperature and Food Availability on Particle Mixing by the Bivalve Abra alba Using New Image Analysis Techniques
Source: PLoS One. 2016 Apr 26;11(4):e0154270. doi: 10.1371/journal.pone.0154270 (PMC4846090; doi:10.1371/journal.pone.0154270)
Supplement: S1 Software — (DOCX) [file pone.0154270.s001.docx]

Supporting Information S1:

The following link can be used to a download the compress file archive containing: (1) a time-lapse sequence of 500 original images (abra_exp3.avi that can be found in the “DemoFilm” folder, (2) a compiled version of our image-analysis programming code (“TracklumS”, you can find technical requirements, installation and execution instructions in the pdf- or txt-file “ReadMe”), (3) a guide on how to use and parametrize this code to visualize data and to reproduce the raw data mentioned below (pdf-file HowTo), and (4) raw data regarding isolated luminophores, waiting times and jump lengths isolated from the above-mentioned time-lapse sequence (in txt format that can be found in the DemoFilm folder, see the pdf-file “HowTo”).

<https://bfs.u-bordeaux.fr/telecharge.php?choix=files/2977df2613f739b7a2e019128d9a1d46/TrackLumS.zip>
